# Cell Volume Regulation in Cultured Human Retinal Müller Cells Is Associated with Changes in Transmembrane Potential

**DOI:** 10.1371/journal.pone.0057268

**Published:** 2013-02-25

**Authors:** Juan M. Fernández, Gisela Di Giusto, Maia Kalstein, Luciana Melamud, Valeria Rivarola, Paula Ford, Claudia Capurro

**Affiliations:** 1 Laboratorio de Biomembranas, Departamento de Fisiología y Biofísica, Facultad de Medicina, Universidad de Buenos Aires, Ciudad Autónoma de Buenos Aires, Buenos Aires, Argentina; 2 Consultorio de Neuroinmunología, Centro Universitario de Neurología Dr. J.M. Ramos Mejía, Facultad de Medicina, Universidad de Buenos Aires, Ciudad Autónoma de Buenos Aires, Buenos Aires, Argentina; Albany Medical College, United States of America

## Abstract

Müller cells are mainly involved in controlling extracellular homeostasis in the retina, where intense neural activity alters ion concentrations and osmotic gradients, thus favoring cell swelling. This increase in cell volume is followed by a regulatory volume decrease response (RVD), which is known to be partially mediated by the activation of K^+^ and anion channels. However, the precise mechanisms underlying osmotic swelling and subsequent cell volume regulation in Müller cells have been evaluated by only a few studies. Although the activation of ion channels during the RVD response may alter transmembrane potential (V_m_), no studies have actually addressed this issue in Müller cells. The aim of the present work is to evaluate RVD using a retinal Müller cell line (MIO-M1) under different extracellular ionic conditions, and to study a possible association between RVD and changes in V_m_. Cell volume and V_m_ changes were evaluated using fluorescent probe techniques and a mathematical model. Results show that cell swelling and subsequent RVD were accompanied by V_m_ depolarization followed by repolarization. This response depended on the composition of extracellular media. Cells exposed to a hypoosmotic solution with reduced ionic strength underwent maximum RVD and had a larger repolarization. Both of these responses were reduced by K^+^ or Cl^−^ channel blockers. In contrast, cells facing a hypoosmotic solution with the same ionic strength as the isoosmotic solution showed a lower RVD and a smaller repolarization and were not affected by blockers. Together, experimental and simulated data led us to propose that the efficiency of the RVD process in Müller glia depends not only on the activation of ion channels, but is also strongly modulated by concurrent changes in the membrane potential. The relationship between ionic fluxes, changes in ion permeabilities and ion concentrations –all leading to changes in V_m_– define the success of RVD.

## Introduction

Glial cells in the sensory retina (Müller cells) are mainly involved in controlling osmotic and ionic homeostasis [1], [2]. During intense neuronal activity, retinal cells can be surrounded by a hypoosmotic environment, since light-evoked changes in the ionic composition of the extracellular fluid cause a decrease in osmolarity, thus favoring glial swelling [3]. In most cell types this increase in cell volume is followed by a regulatory volume decrease response (RVD) partially mediated by the activation of K^+^ and anion channels [4], [5], [6]. However, only a few studies have evaluated the mechanisms underlying cell volume regulation in Müller cells [7], [8]. It has been reported that Müller cells *in situ* show an effective control of cell volume, that prevents cell swelling, probably due to the presence of K^+^ channels K_ir_ 4.1. The expression of these channels is altered in different pathologies such as retinal ischemia, ocular inflammation and diabetes, as well as in organ cultures [9], [10], [11], [12].

Changes in the extracellular ion composition of the retina during neural activity also cause changes in transmembrane potential (V_m_) and in the chemical gradients of most of the ions that determine RVD. In addition, the activation of ion channels during RVD may also alter V_m_. However, to our knowledge, no studies have investigated the putative link between cell volume regulation and V_m_ in these cells.

The channels involved in the RVD response have been studied in different cell types, usually by evaluating changes in cell volume with and without blockers. The identification and characterization of these channels is typically performed through excised or whole cell patch clamp studies [13], [14], [15]. Though these methods undeniably offer important and reliable information on conductance changes during cell swelling, they fail to do so during cell volume regulation, since they do not preserve cell membrane integrity nor intracellular medium composition. This could explain the reason why only a few reports have been able to evaluate the RVD response in a more physiological context [16], [17], [18].

The aim of the present work is to characterize, for the first time, the RVD response in a retinal Müller cell line (MIO-M1) under different extracellular ionic conditions and to evaluate a possible association between RVD and changes in V_m_. Cell volume and V_m_ changes were measured using fluorescent probe techniques. We also developed a mathematical model that provides information on electrochemical ion gradients and solutes fluxes during the RVD response.

Our results show that cell swelling and subsequent RVD is accompanied by V_m_ depolarization followed by repolarization. However, this RVD response depends closely on the composition of extracellular media. Although K^+^ and Cl^−^ channels do play an important role in the RVD response of these cells, their contribution is evident only if a significant driving force for KCl efflux is present.

## Materials and Methods

### Cell Cultures

The MIO-M1 cell line (kindly provided by Dr. Astrid Limb, University College London, London, UK) is a spontaneously immortalized retinal Müller glial cell line, originated from human retina, that retained many characteristics of Müller cells [19]. Cells were grown as monolayers in the presence of Dulbecco’s Modified Eagle Medium (DMEM)/glutamax supplemented with 10% fetal calf serum (FCS), containing 5 µg/ml streptomycin and 5 U/ml penicillin at 37°C in a humidified atmosphere containing 5% CO_2_. Cells were routinely subcultured every week, and those to be studied were grown on coverslips during 3–4 days before recording.

### Solutions and Chemicals

Two different isoosmotic solutions were employed: 1- NaCl solution (ISO_NaCl_) and 2- Mannitol solution (ISO_Mannitol_), in which 50 mM NaCl were replaced with 100 mM Mannitol (Table 1). Hypoosmotic solutions were prepared from each isoosmotic solution by the removal of either NaCl (HYPO_NaCl_) or Mannitol (HYPO_Mannitol_), thus varying ion composition in the first case, or keeping it constant in the second (Table 1). Cells were set in an external isoosmotic solution for at least 10 minutes, and then a hypoosmotic shock was induced. All solutions were titrated to pH 7.40 using NaOH (Sigma-Aldrich), and osmolalities were routinely measured by a pressure vapor osmometer (Wescor).

**Table 1:** Ionic composition of experimental external solutions.

Composition	ISO_NaCl_	HYPO_NaCl_	ISO_Mannitol_	HYPO_Mannitol_
NaCl (mM)	126	76	70	70
KCl (mM)	5.5	5.5	5.5	5.5
CaCl_2_ (mM)	2.5	2.5	2.5	2.5
MgCl_2_ (mM)	1.25	1.25	1.25	1.25
Hepes (mM)	20	20	20	20
Glucose (mM)	10	10	10	10
Mannitol (mM)	0	0	100	0
Osmolarity (mOsM, 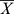 ±SEM)	299±2	200±2	293±2	195±1

In some experiments 10^−3^ M BaCl_2_ (vehicle: water) or 10^−4^ M 5-Nitro-2-(3-phenylpropylamino) benzoic acid (NPPB; vehicle: DMSO) were added to iso- and hypoosmotic solutions, and cells were pre-incubated in isoosmotic extracellular solutions containing blockers or vehicles for 10 minutes.

2′,7′-Bis(2-carboxyethyl)-5(6)-carboxyfluorescein-acetoxymethylester (BCECF-AM, 3.2 mM, Molecular Probes) and bis-(1,3-dibutylbarbituric acid)trimethine oxonol (DIBAC4_(3)_, 0.6 mM, Molecular Probes) stock solutions were dissolved in DMSO and stored at −20°C until used.

### Measurement of Cell Volume Changes and RVD Response

MIO-M1 cells grown on coverslips were mounted on a chamber and loaded with 6 µM BCECF-AM for 30 minutes at 20°C. The chamber was then placed on the stage of a Nikon TE-200 epifluorescence inverted microscope (Nikon Planfluor 40X oil immersion objective lens) as previously described [20]. Fluorescence intensity was recorded by exciting BCECF at the isosbestic point (440 nm), where the fluorochrome is pH insensitive. Fluorescence data were acquired every 10 seconds using a charge coupled device camera (Hamamatsu C4742-95) connected to a computer with the Metafluor acquisition program (Universal Imaging Corporation, PA). The procedure used to estimate cell water volume was similar to the one previously described [20], [21]. Changes in cell volume can be calculated as follows:
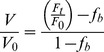
(1)where *V* is cell volume at time *t*; *V_0_* is cell volume at t* = *0; *F_0_* represents the signal obtained from a small region of the cell (pinhole), when placed in equilibrium with an isoosmotic medium with an osmolality OsM_0_; *F_t_* is the fluorescence from the same region at time *t*, when placed in equilibrium with a solution with an osmolality of OsM_t_ and *f_b_* is the relative background. This parameter corresponds to the *y* intercept of a plot of *F_t_/F_0_* versus OsM_0_/OsM_t_, which represents relative fluorescence in the absence of osmolality changes.

RVD after cell exposure to a hypoosmotic medium was calculated by the following equation:
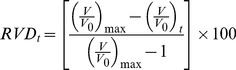
(2)where *(V/V_0_)_max_* is the maximal value of V/V_0_ attained during hypoosmotic swelling (peak), and *(V/V_0_)_t_* represents the value of V/V_0_ observed at time *t. RVD_t_* thus denotes the magnitude of volume regulation at time *t*, with 100% RVD indicating complete volume regulation and 0% RVD indicating no volume regulation.

### Measurement of Membrane Voltage Changes

**Figure 1:**
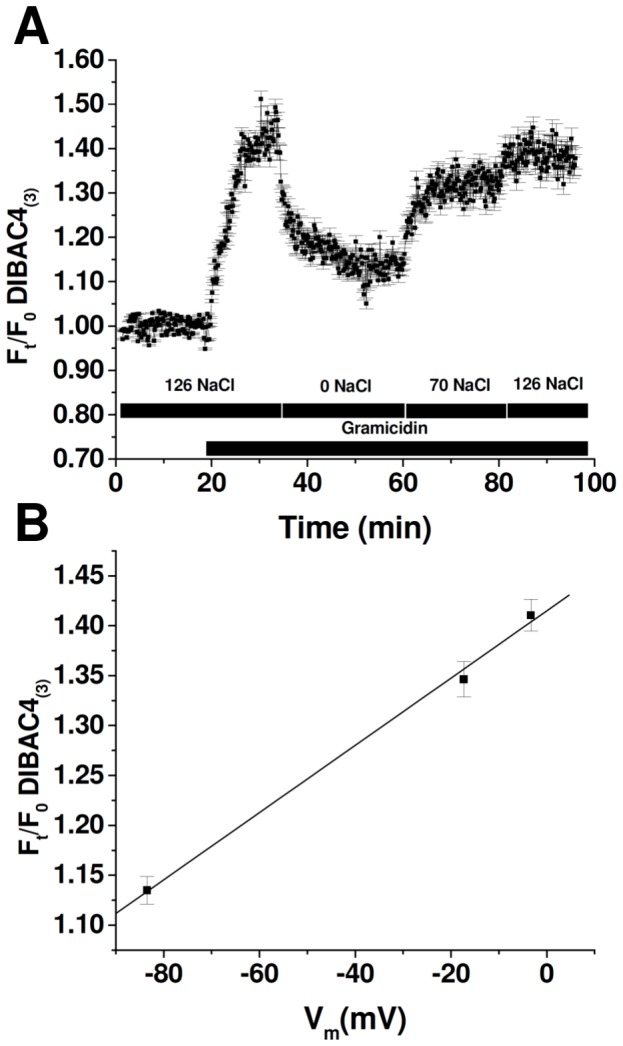
Calibration of voltage sensitive dye DIBAC4_(3)_ in MIO-M1 cells. **A-** Representative experiment showing the response of cells previously loaded with 2.5 µM DIBAC4_(3)_ for 15 minutes, exposed to different extracellular concentrations of NaCl. Points represent changes in fluorescence intensity relativized to the stationary values, in the absence of gramicidin (F_t_/F_0_ DIBAC4_(3)_). When a stable signal was registered, control solution was replaced by a solution containing 5 µM gramicidin. Afterwards, extracellular NaCl concentration was replaced (0 mM, 70 mM and 126 mM). **B-** Relation between relative changes in fluorescence and membrane potential calculated from Equation 3.

Transmembrane potential was measured using DIBAC4_(3)_, a slow response anionic dye which emission has previously been shown to be independent of cell volume changes [22]. The intracellular concentration of DIBAC4_(3)_ depends on V_m_ following a Nernstian distribution [23], [24]. Cells were loaded with 2.5 µM DIBAC4_(3)_ for 15 minutes at 20°C and placed on the stage of the same microscope described in the previous section. Excitation wavelength was 490 nm. Emitted light (above 520 nm) was recorded at 10 second intervals. Fluorescence intensity was monitored until it reached a stable value before starting the experiments. Fluorescence intensity changes after interventions were relativized to stationary values (F_t_/F_0_) and data were corrected for background noise and drift. Calibration was made by adding 5 µM gramicidin and 2.5 µM DIBAC4_(3)_ to solutions containing different concentrations of NaCl replaced with N-Methyl-D-glucamine chloride (NMDGCl) (Figure 1). V_m_ was calculated as:
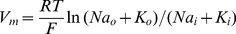
(3)


Intracellular concentrations of Na^+^ and K^+^ where assumed to be 18 mM and 132 mM, respectively. Three extracellular NaCl concentrations were tested, 126 mM, 70 mM and 0 mM, corresponding to membrane potentials of −3.32 mV, −17.33 mV and −83.47 mV, respectively. A 1% change in fluorescence corresponds to a V_m_ variation of 2.2 mV, as calculated from the mean calibration curve (0.0045±0.002, 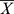
±SD, n = 58).

### Osmotic Swelling and RVD Response Modeling

Cell volume and V_m_ changes were simulated by using a mathematical model that implies the following assumptions:

1. Cells are non-polarized and cell membrane surface area available for solute and water transport (A_c_) remains constant, regardless of cell volume changes.
2. The composition of extracellular solution remains constant, unless the solution is changed to induce an osmotic shock. Cell swelling occurs due to transmembrane water flux driven by osmotic gradients across the membrane.
3. Intracellular osmolarity is determined by the sum of Na^+^, K^+^, Cl^−^, and impermeant anions (X_i_) concentrations. The value of the mean charge (z) of X_i_ is assumed to be the one that fulfils the electroneutral condition (≅−1).
4. The membrane contains channels that allow the diffusive flux of ions (Na^+^, K^+^, and Cl^−^ channels) and water (Aquaporins). To achieve a stationary condition, passive ion fluxes are assumed to be compensated by opposing active fluxes that do not balance those generated during RVD. Active fluxes were assumed to be constant since several previous reports demonstrated that short-term volume regulation is not affected by them [25], [26].
5. RVD response is achieved by an increase in K^+^ and/or Cl^−^ permeabilities with a latency (τ) that was arbitrarily determined to be 20 s after the initiation of swelling. Increases in ion permeabilities develop in a time-dependent exponential manner according to:

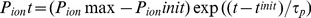
(4)Where P*_ion_*t is ion permeability at time *t*, P*_ion_*max is the maximum permeability achieved during RVD and P*_ion_*init is the initial permeability (Table 2). t^init^ is the time at which permeability changes start and τ_p_ is a time constant (90 s) [27].

**Table 2:** Values of parameters used in simulations.

Parameter	NaCl	Mannitol	Units
***Cell Parameters***			
V_cell_	1×10^−8^	0.945×10^−8^	cm^3^
A_c_	2×10^−5^	2×10^−5^	cm^2^
P_Na_	1××10^−7^	1×10^−7^	cm.s^−1^
P_K_	2×10^−6^	2×10^−6^	cm.s^−1^
P_K_max	6×10^−6^	6×10^−6^	cm.s^−1^
P_Cl_	5×10^−7^	5×10^−7^	cm.s^−1^
P_Cl_max	5×10^−6^	5×10^−6^	cm.s^−1^
P_f_	1.5×10^−2^	1.5×10^−2^	cm.s^−1^
[Na]_i_	0.184×10^−4^	0.195×10^−4^	mol.cm^−3^
[K]_ i_	1.32×10^−4^	1.31×10^−4^	mol.cm^−3^
[Cl]_ i_	0.291×10^−4^	0.226×10^−4^	mol.cm^−3^
[X]_ i_	1.10×10^−4^	1.16×10^−4^	mol.cm^−3^
V_m_	−6.31×10^−2^	−6.71×10^−2^	Volts
τ	20	20	s (post-shock)
***Isosmotic Solution***			
[Na]_o_	1.26×10^−4^	0.76×10^−4^	mol.cm^−3^
[K]_o_	0.055×10^−4^	0.055×10^−4^	mol.cm^−3^
[Cl]_o_	1.315×10^−4^	0.815×10^−4^	mol.cm^−3^
[X]_o_	0.27×10^−4^	1.27×10^−4^	mol.cm^−3^
***Hyposmotic Solution***			
[Na]_o_	0.76×10^−4^	0.76×10^−4^	mol.cm^−3^
[K]_o_	0.055×10^−4^	0.055×10^−4^	mol.cm^−3^
[Cl]_o_	0.82×10^−4^	0.815×10^−4^	mol.cm^−3^
[X]_o_	0.27×10^−4^	0.27×10^−4^	mol.cm^−3^

Data were obtained either from our own measurements in MIO-M1 cells or from literature, as follows: i- V_cell_ and A_c_ in isotonic conditions were estimated from confocal images as previously reported [43]; ii- P_f_ was obtained from the measured cell volume changes during a hypoosmotic challenge using a modification of the Fick’s law [20]; iii- V_m_ corresponds to the value recorded using sharp electrode patch clamp technique by Limb et al [19]; iv- intracellular Na^+^, K^+^ and Cl^−^ concentration are the typical values for most cell types; v- extracellular Na^+^, K^+^ and Cl^−^ concentrations are the same as in experimental solutions; vi- ion permeabilities were chosen in order to obtain the V_m_ value of MIO-M1 cells; vii- the initial parameters presented for the Mannitol condition were obtained by simulating an extracellular solution change, from ISO_NaCl_ to ISO_Mannitol_ (see Materials and Methods section).

#### Mathematical model

Considering the assumptions indicated above, and given the initial values detailed in Table 2, the values for intracellular Na^+^ mass (m_Na_), K^+^ mass (m_K_), Cl^−^ mass (m_Cl_), osmotically active cell volume (V_cell_) and V_m_ were computed at each iteration step (0.1 seconds). Equilibrium potentials (Eq) were calculated by using the Nernst equation:
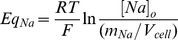


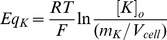
(5)

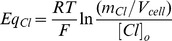



Where *R* is the gas constant, *T* is the absolute temperature in Kelvin, *F* is the Faraday constant and *[Ion]_o_* is the external concentration of the ion in question.

V_m_ was determined by the Goldman-Hodgkin-Katz equation:
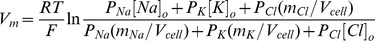
(6)


Diffusive fluxes (J_ion_) were given by [28], [29]:
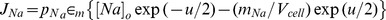


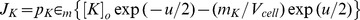
(7)

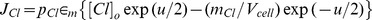
Where 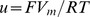, and 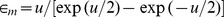, and a negative value of *J*_ion_ indicates an outward flux. Furthermore, the time courses of m_Na_, m_K_ and m_Cl_ were given by:
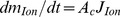
(8)And from Fick’s law of diffusion, the variation rate of Vcell is:
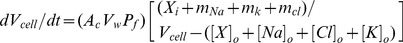
(9)Where Vw is the partial molar volume of water, Pf is the osmotic water permeability of the membrane and [X]o is the concentration of external impermeable solutes.

#### Numerical methods and simulation conditions

Equations 8 and 9 were integrated numerically by the Euler method with a time step of 0.1 seconds. Total simulated time was 2,400 seconds. At each iteration step, V_m_ and Eq for Na^+^, K^+^, and Cl^−^ were calculated by equations 6 and 5, respectively. External osmolarity was made hypoosmotic by two different approaches: 1- Reducing NaCl concentration (HYPO_NaCl_), or 2- Reducing the concentration of external impermeable solutes (X_o_, e.g. Mannitol, HYPO_Mannitol_). Afterwards, both conditions were simulated (Table 2):

NaCl: External isoosmotic solution contains 1.26×10^−4^ mol.cm^−3^ NaCl (290 mOsM). Hypoosmotic shock was achieved by reducing external NaCl concentration to 0.76×10^−4^ mol.cm^−3^ (190 mOsM).

Mannitol: Initial parameters were obtained by simulating the change of extracellular solutions, from ISO_NaCl_ to ISO_Mannitol_. In this condition, external isoosmotic solution contains 0.76×10^−4^ mol.cm^−3 ^NaCl and 1.27×10^−4^ mol.cm^−3^ of an impermeable non-charged solute (X_o_) (290 mOsM). Hypoosmotic shock was achieved by reducing external X_o_ concentration to 0.27×10^−4^ mol.cm^−3^ (190 mOsM).

Once external solutions were changed, cells were subjected to RVD activation after a latency (τ) of 20 seconds (post solution change).

### Statistics

Values are reported as mean ± SEM, and n is the number of cells evaluated in each condition. Student’s t Test for unpaired data was used according to the protocol; p<0.05 was considered a significant difference.

## Results

### Effect of Extracellular Media Composition on RVD Response in MIO-M1 Cells

**Figure 2:**
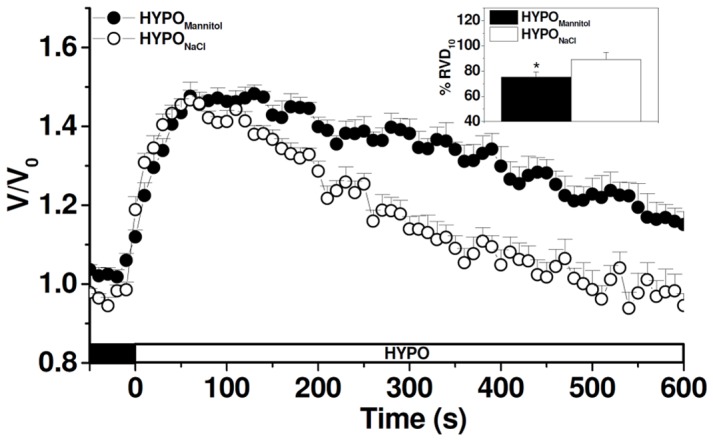
Effects of extracellular media composition on RVD in MIO-M1 Cells. Representative kinetics of cell volume changes measured in BCECF-loaded MIO-M1 cells in response to hypoosmotic shock (ΔOsM = 100 mOsM) generated either by varying (HYPO_NaCl_) or keeping constant extracellular ion composition (HYPO_Mannitol_). Insert: Percentage of cell volume recovery at 10 minutes (% RVD_10_) in both conditions. Values are mean ± SEM for 42–55 cells from 15 experiments, *p<0.05, HYPO_Mannitol_ vs. HYPO_NaCl_.

We first characterized RVD response in MIO-M1 cells exposed to a hypoosmotic shock, generated, either varying or keeping constant ion composition (HYPO_NaCl_ or HYPO_Mannitol_, respectively). Figure 2 shows the time course of relative cell volume changes (V/V_0_) in response to these hypoosmotic gradients (ΔOsM = 100 mOsM). Though in both conditions cells respond to the hypoosmotic challenge with rapid swelling and thereafter exhibit RVD, kinetics were quite different in each case. In cells faced with HYPO_NaCl_, cell volume is restored more rapidly, as compared to HYPO_Mannitol_ experiments. Indeed, the percentage of RVD at 10 minutes (% RVD_10_) is significantly higher with the HYPO_NaCl_ solution (Figure 2 insert). These results indicate that although MIO-M1 cells respond to cell swelling by triggering RVD, the magnitude of this response depends on extracellular media composition.

**Figure 3:**
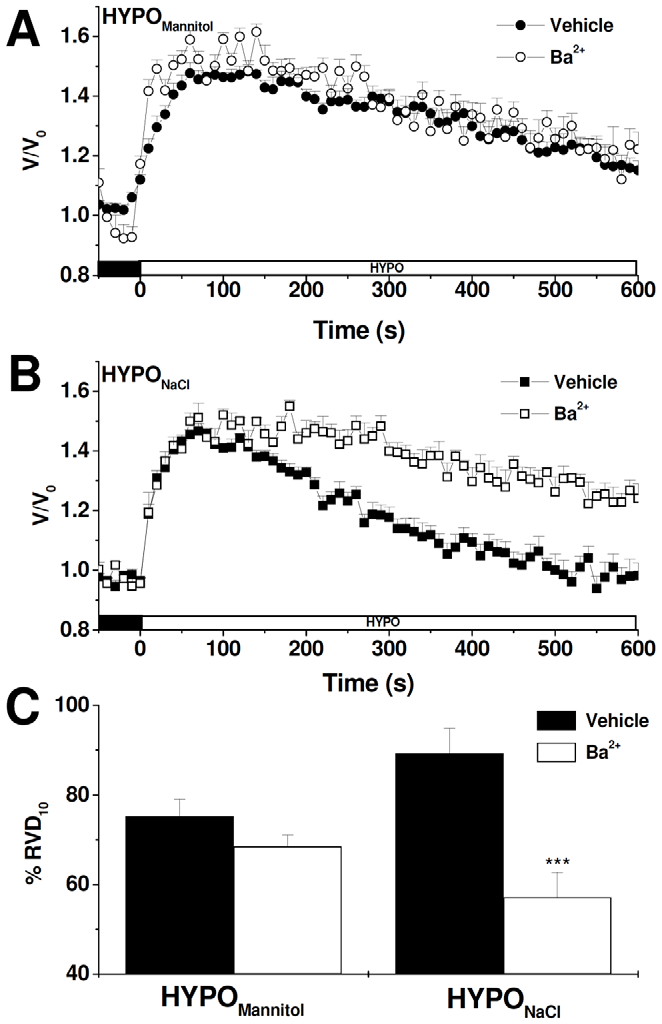
Role of Ba^2+^- sensitive K^+^ channels on RVD in MIO-M1 Müller cells. Representative cell volume changes measured in BCECF-loaded MIO-M1 cells in response to a hypoosmotic shock (ΔOsM = 100 mOsM) generated either keeping constant (HYPO_Mannitol_) (**A**) or varying ion composition (HYPO_NaCl_) (**B**). In all the experiments 10^−3^ M Ba^2+^ or vehicle (water) was added to ISO_NaCl_ or ISO_Mannitol_ 10 minutes before the hypoosmotic shock and maintained during the entire experiment. **C-** % RVD_10_ after the hypoosmotic challenge in vehicle or Ba^2+^ treated cells. Values are mean ± SEM for 21–80 cells from 5–9 experiments, ***p<0.001, Vehicle vs. Ba^2+^.

**Figure 4:**
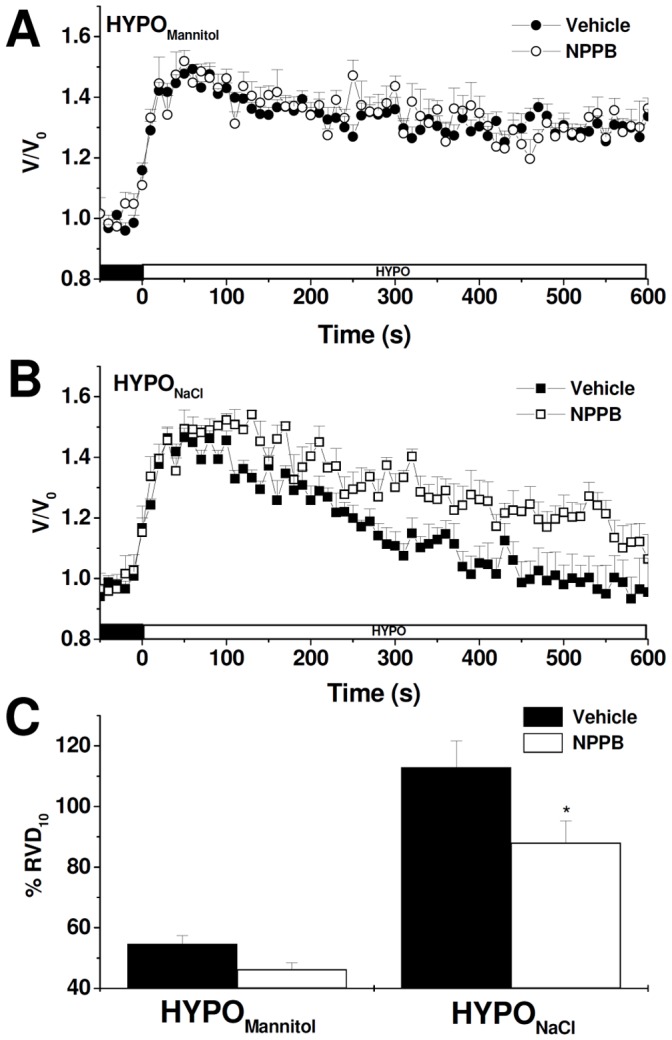
Role of NPPB-sensitive Cl^−^ channels on RVD in MIO-M1 cells. Representative cell volume changes measured in BCECF-loaded MIO-M1 cells in response to a hypoosmotic shock (ΔOsM = 100 mOsM) generated either keeping constant (HYPO_Mannitol_) (**A**) or varying ion composition (HYPO_NaCl_) (**B**). In all the experiments 10^−4^ M NPPB or vehicle (DMSO) was added to ISO_NaCl_ or ISO_Mannitol_ 10 minutes before the hypoosmotic shock and maintained during the entire experiment. **C-** % RVD_10_ after the hyposmotic challenge in DMSO or NPPB treated cells. Values are mean ± SEM for 28–76 cells from 5–13 experiments, *p<0.05, Vehicle vs. NPPB.

Since RVD is known to be attained by the activation of K^+^ and Cl^−^ conductances in most mammalian cell types [30], we further investigated RVD response in the presence of volume-sensitive Cl^−^ channels blockers (NPPB) or K^+^ channels blockers (Ba^2+^), using cells exposed alternatively to either external media. Figure 3 illustrates that, in the presence of Ba^2+^, the time course of relative cell volume changes is not affected in MIO-M1 cells exposed to HYPO_Mannitol_ (A) while it is significantly retarded in cells faced with HYPO_NaCl_ (B). Thus, RVD is significantly decreased by Ba^2+^ only when external NaCl concentration varies (HYPO_NaCl_) (C). Similar results are observed in the presence of the volume-sensitive Cl^−^ channels blocker NPPB (Figure 4A–C). These results clearly indicate that K^+^ and Cl^−^ channels are involved in the RVD response of MIO-M1 cells; however, their participation is evident only under certain experimental conditions.

### Effect of Extracellular Medium Composition on V_m_ during a Hypoosmotic Challenge in MIO-M1 Cells

**Figure 5:**
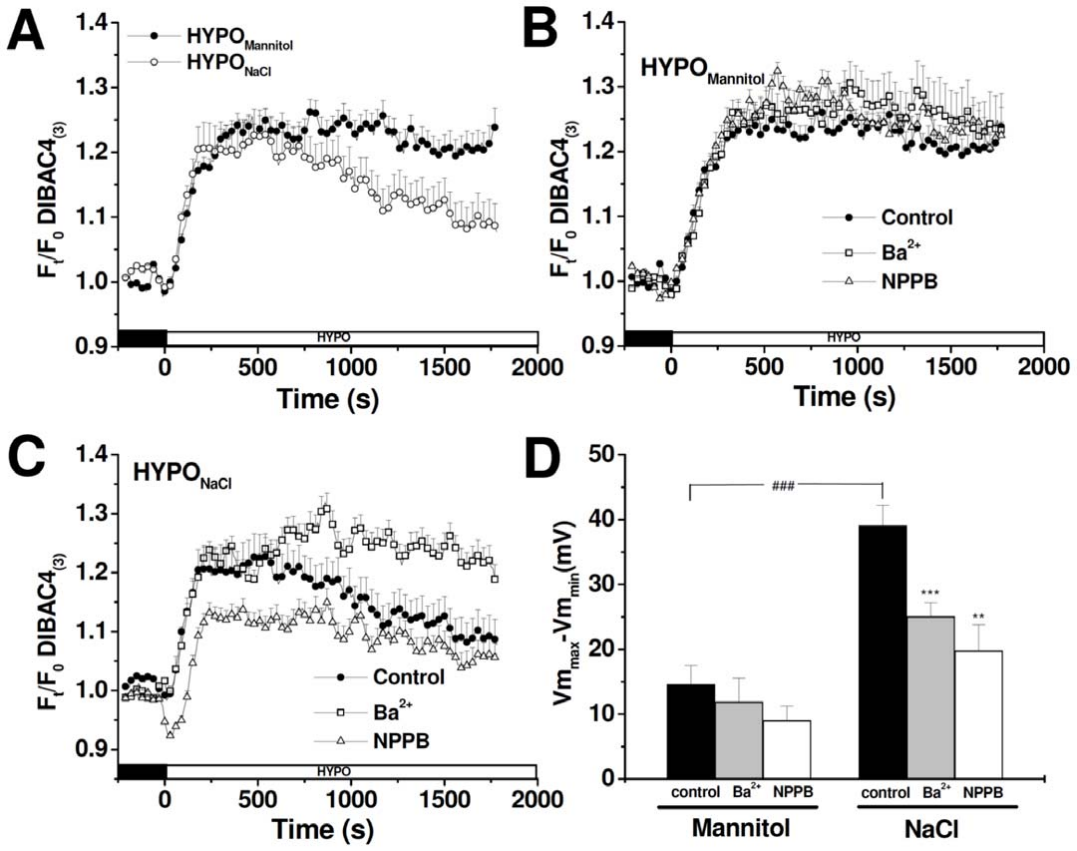
V_m_ evolution after a hypoosmotic shock in MIO-M1 cells. V_m_ was monitored using DIBAC4_(3)_ under different experimental conditions. **A–**V_m_ changes measured in response to a hypoosmotic shock (ΔOsM = 100 mOsM) generated either by varying (HYPO_NaCl_) or keeping constant ion composition (HYPO_Mannitol_). Effect of 10^−3^ M Ba^2+^ and 10^−4^ M NPPB on V_m_ changes under HYPO_Mannitol_ (**B**) or under HYPO_NaCl_ conditions (**C**). **D-** Bars indicating the difference between the peak maximum V_m_ and the V_m_ 30 minutes after being exposed to a hypoosmotic media (Vm_max_−Vm_min_) obtained after the hypoosmotic shock under each experimental condition. This value indicates the degree of repolarization after the initial swelling-induced depolarization. Values are mean ± SEM for 21–46 cells from 3–7 experiments, ###p<0.001, NaCl vs. Mannitol; ***p<0.001, Ba^2+^ vs. Control, **p<0.01, NPPB vs. Control.

In this set of experiments, we evaluated V_m_ after a hypoosmotic shock (ΔOsM = 100 mOsM) generated either varying or keeping a constant extracellular ion composition, and using the potentiometric dye DIBAC4_(3)_. Figure 5A shows the time course of fluorescence changes (F_t_/F_0_). The response in both conditions consists of an initial depolarization followed by a partial repolarization. The magnitude of this repolarization is assessed as the difference between peak maximum V_m_ and V_m_ 30 minutes after exposure to a hypoosmotic media (Vm_max_−Vm_min_). As seen in Figure 5D, repolarization is significantly larger in the HYPO_NaCl_ condition than in the HYPO_Mannitol_ condition.

We then evaluated whether Ba^2+^ or NPPB treatments affect V_m_ before and after the hypoosmotic shock. When Ba^2+^ is added to cells exposed to isoosmotic solutions (Mannitol or NaCl), V_m_ depolarizes (ΔV_m = _50±3 mV, n = 57), thus indicating that Eq_K_ is negative in relation to V_m_. In contrast, the addition of NPPB induces hiperpolarization of V_m_ (ΔV_m_ = −53±1.4 mV, n = 128), thus indicating that Eq_Cl_ is positive in relation to V_m_. These changes in resting V_m_ suggest that Ba^+2^-sensitive K^+^ channels and NPPB-sensitive Cl^−^ channels contribute to resting potential. Interestingly, after the osmotic shock, the presence of Ba^2+^ or NPPB does not affect the magnitude of repolarization in HYPO_Mannitol_, but does significantly reduce V_m_ repolarization in the HYPO_NaCl_ condition (Figure 5B–D).

These results, together with those from the previous section, suggest that there may be an interplay between RVD and V_m_ repolarization, and that both depend on extracellular media composition.

### Simulation of Cell Volume and V_m_ Changes under Different Extracellular Ion Composition

A mathematical model was designed to investigate why RVD and V_m_ changes differ between cells exposed to a hypoosmotic challenge (ΔOsM = 100mOsM) by varying NaCl composition or by removing mannitol (and thus unchanging NaCl composition). The model considers two different conditions: one in which cells face the HYPO_NaCl_ extracellular solution and another in which cells are exposed to the HYPO_Mannitol_ extracellular solution (Table 2). We calculated V_cell_ changes, V_m_ and E_q_ as well as net ionic flux (J_net_) under both conditions. Since RVD depends on the activation of K^+^ and Cl^−^ channels, a regulatory response was simulated by increasing both ion permeabilities at t = 20 s.

**Figure 6:**
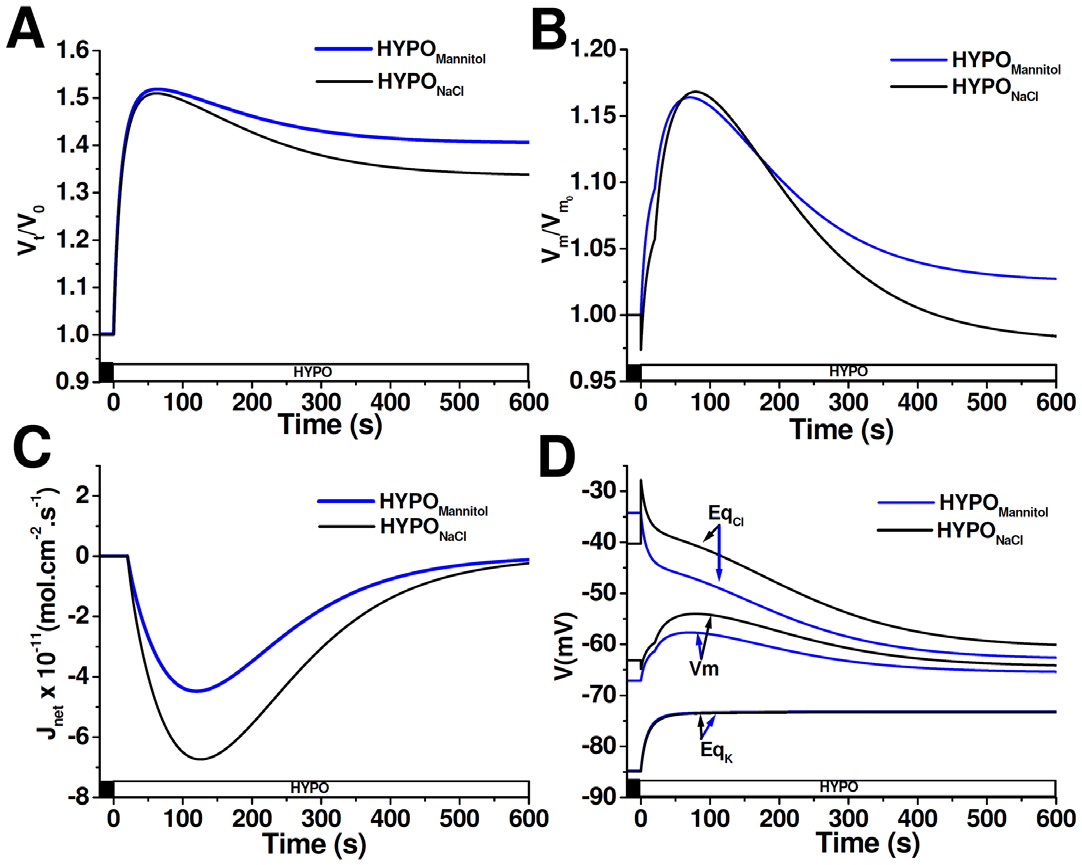
Modeling of cells exposed to different extracellular media compositions. Time courses of V_t_/V_0_ (**A**), V_m_/V_m0_ (**B**), J_net_ (**C**) and V_m_, Eq_Cl_, Eq_K_ (**D**) simulated in cells exposed to either HYPO_NaCl_ or to HYPO_Mannitol_. At time = 0 extracellular osmolarity was reduced (ΔOsM = 100 mOsM) and after a delay of 20 s, P_K_ and P_Cl_ increased according to Equation 4. A negative value of *J*_net_ indicates an outward flux.

Figure 6A shows that after the hypoosmotic challenge, cells exposed to HYPO_NaCl_ initially swell and then partially restore their original volume reaching a new steady-state (%RVD_10_ 34%). A similar response is observed in cells exposed to HYPO_Mannitol_, but their volume is re-established to a lesser extent (24%, Figure 6A). Simulated relative changes in V_m_ show that the difference in %RVD_10_ among both conditions is associated with a disparity in the magnitude of repolarization (Figure 6B). As expected, under both conditions, Cl^−^ exiting the cell is electrically coupled to K^+^ efflux, thus leading to a *quasi* electroneutral KCl efflux followed by water. The reduced %RVD_10_ in HYPO_Mannitol_, as compared to HYPO_NaCl,_ is due to a reduced net osmolyte efflux (Figure 6C). Net osmolyte efflux, and therefore RVD, ends just when V_m_ reaches a new stationary value. The evolution of equilibrium potentials and V_m_ during these simulations is illustrated in Figure 6D. Since in the HYPO_NaCl_ condition hypoosmolarity is achieved by removing NaCl, immediately after the hypoosmotic shock, [Cl]_o_ suddenly decreases, transiently increasing the equilibrium potential of this ion (Figure 6D, t = 0, peak Eq_Cl_). The subsequent swelling determines a depolarization associated with the dilution of intracellular K^+^. Since in this cell Cl^−^ exhibits an equilibrium potential that is positive as compared to V_m_, when RVD is activated at 20 s, an additional increase in V_m_ is produced due to the opening of Cl^−^ channels. As intracellular Cl^−^ concentration decreases, Eq_Cl_ becomes more negative. In addition, K^+^ permeability is also increased, a fact that tends to bring V_m_ closer to Eq_K_. As a consequence, V_m_ partially repolarizes during RVD (Figure 6B and 6D).

As opposed to the HYPO_NaCl_ condition, cells exposed to HYPO_Mannitol_ keep extracellular NaCl composition constant; therefore, at the instant that extracellular solution is changed, the concentrations of external and internal permeable ions remain constant. Thus, the substitution of the extracellular solution does not affect *per se* neither Eq_Cl_ nor V_m_ (Figure 6D, t = 0 s, Eq_Cl_). During cell swelling, the concentration of all intracellular species is reduced, which explains the increase of Eq_K_ and the decrease of Eq_Cl_ (Figure 6D, t = 0–20 s). Mainly, it is the dilution of intracellular Cl^−^, together with the initial depolarization due to the opening of volume-activated Cl^−^ channels, what reduces the electrochemical gradient for Cl^−^. As a consequence, V_m_ repolarization and cell volume regulation are smaller than in the HYPO_NaCl_ condition.

**Figure 7:**
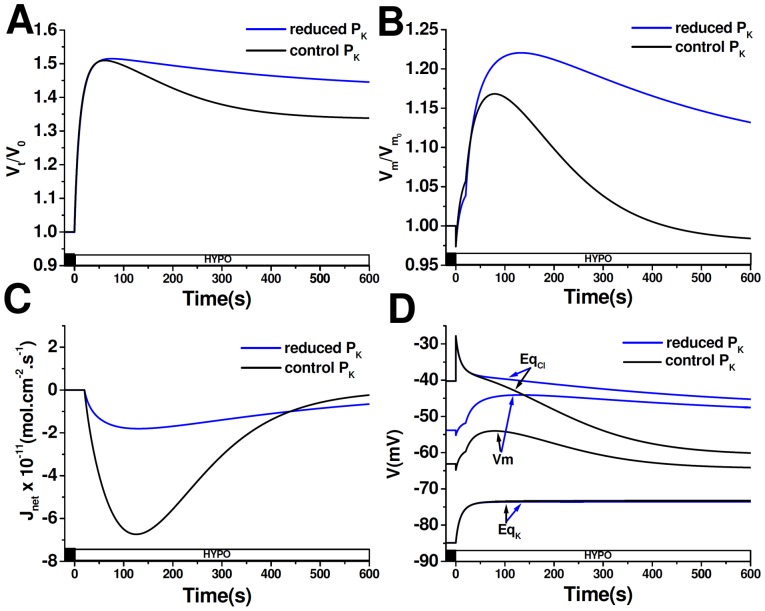
Modeling of cell response to HYPO_NaCl_ when P_K_ is reduced. Time courses of V_t_/V_0_ (**A**), V_m_/V_m0_ (**B**), J_net_ (**C**) and V_m_, Eq_Cl_, Eq_K_ (**D**) simulated in cells exposed to HYPO_NaCl_. Before the hypoosmotic shock, resting P_K_ was reduced by half and remained constant throughout the entire simulation. At time = 0 extracellular osmolarity was reduced (ΔOsM = 100 mOsM) and after a delay of 20 s, P_Cl_ −but not P_K_− was increased, according to Equation 4. A negative value of *J*_net_ indicates an outward flux.

Given that our experimental results showed that the reduction of RVD by K^+^ and Cl^−^ channels blockers is only evident in cells exposed to HYPO_NaCl_, the following simulations were performed in this condition. Figure 7 shows the time course of V_t_/V_0_, Eq_Cl_, Eq_K_, V_m_ and J_net_ when K^+^ permeability is decreased (reduced P_K_), thus mimicking cells exposed to Ba^2+^, versus control conditions (control P_K_). Experimental data indicate that the blockage of K^+^ channels with Ba^2+^ in MIO-M1 cells has two effects: 1) When added to an isoosmotic solution, V_m_ depolarizes and 2) When added in the presence of a hypoosmotic shock, it significantly reduces RVD response. Simulations take into account these effects and therefore, even before the hypoosmotic shock, P_K_ is reduced by half, remaining constant throughout the entire simulation time. Figure 7A shows that when cell P_K_ is reduced, RVD response is lower as compared to control P_K_ (%RVD_10_∶13% vs. 34%, respectively). Even more, a reduced P_K_ leads to a decrease in V_m_ repolarization (Figure 7B) together with a decrease in net osmolyte efflux (Figure 7C). In addition, Figure 7D shows that Cl^−^ electrochemical gradient is reduced while K^+^ electrochemical gradient tends to increase. However, this rise in K^+^ driving force does not contribute to RVD, since P_K_ is reduced.

**Figure 8:**
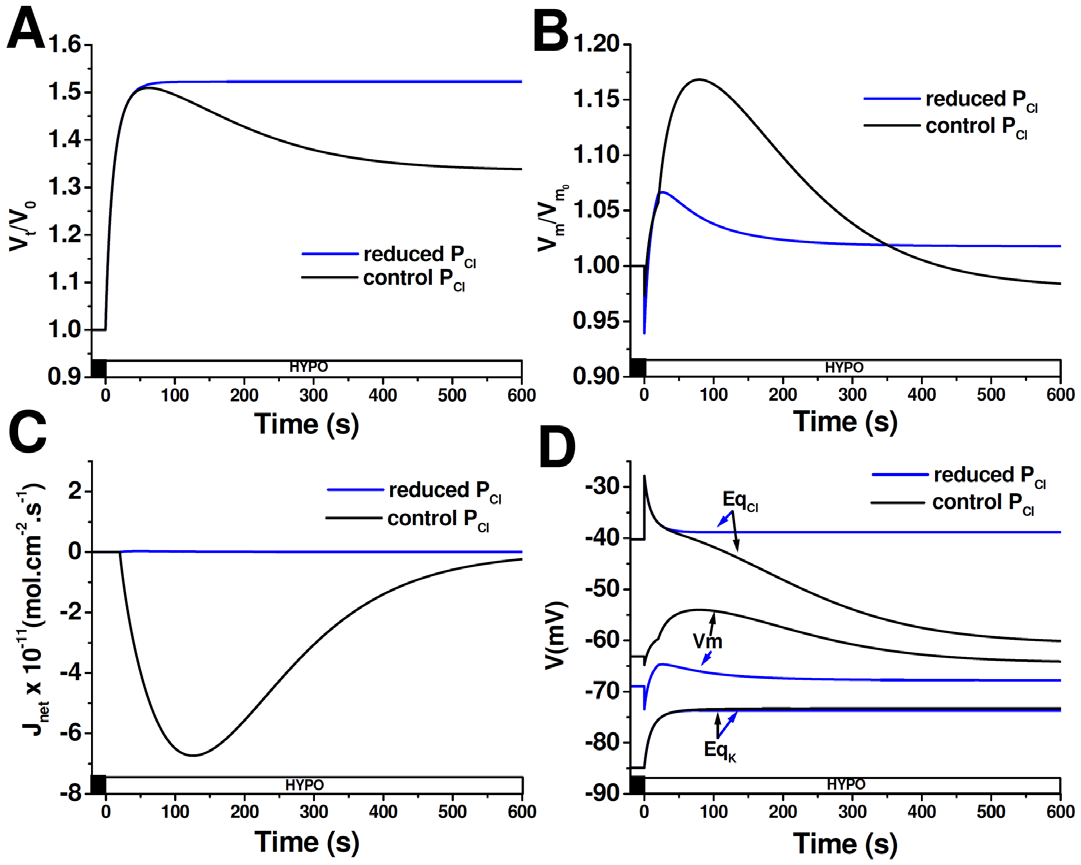
Modeling of cell response to HYPO_NaCl_ when P_Cl_ is reduced. Time courses of V_t_/V_0_ (**A**), V_m_/V_m0_ (**B**), J_net_ (**C**) and V_m_, Eq_Cl_, Eq_K_ (**D**) simulated in cells exposed to HYPO_NaCl_. Before the hypoosmotic shock, resting P_Cl_ was reduced a tenfold and remained constant throughout the entire simulation. At time = 0 extracellular osmolarity was reduced (ΔOsM = 100 mOsM) and after a delay of 20 s, P_K_ −but not P_Cl_− was increased, according to Equation 4. A negative value of *J*_net_ indicates an outward flux.

Figure 8 shows the time course simulations of all the variables described above, when Cl^−^ permeability is decreased (reduced P_Cl_), thus imitating cells exposed to NPPB, versus control conditions (control P_Cl_). Since treatment with NPPB leads to hyperpolarization and to a decrease in the RVD response, these effects are reproduced in our simulations. Before the hypoosmotic shock, P_Cl_ is lowered tenfold and remains constant throughout the simulation. Simulations prove that when cells have a reduced P_Cl_, RVD response is completely absent, V_m_ repolarization is significantly reduced and net osmolyte efflux is almost abolished (Figure 8A–C). Figure 8D shows that, as a consequence of P_Cl_ reduction, resting V_m_ is hyperpolarized and the magnitude of the initial depolarization is diminished. Therefore K^+^ electrochemical gradient is reduced throughout the entire simulation. On the contrary, Cl^−^ electrochemical gradient is augmented; however, this increase in Cl^−^ driving force cannot lead to successful RVD due to the low P_Cl_.

## Discussion

In the present work we evaluated, for the first time, the RVD response in the immortalized retinal Müller cell line, MIO-M1, which maintains important functional characteristics of Müller cells [19]. However, since Müller cells function *in vivo* strongly depends on tissue structure and on the presence of other cell types [31], the extrapolation of our results to the *in vivo* condition may be limited. Nevertheless, taking in mind the appropriate considerations, cell culture is a simple and useful model to get insight into the complex machinery used by Müller cells to regulate their volume.

We showed that MIO-M1 cells respond to a hypoosmotic challenge with cell swelling and subsequent RVD −which is at least in part mediated by K^+^ and Cl^−^ channels− and that this process is associated to V_m_ depolarization followed by repolarization. Our results demonstrate that RVD response depends on the composition of extracellular media. Cells exposed to a hypoosmotic solution with reduced ionic strength (HYPO_NaCl_) underwent maximum RVD (∼100%) and had a larger repolarization. Both of these responses were reduced by K^+^ or Cl^−^ channel blockers. On the other hand, cells facing a hypoosmotic solution with the same ionic strength (HYPO_Mannitol_) as the isoosmotic solution showed a lower RVD (∼75%) and a smaller repolarization and were not affected by blockers. Our mathematical model qualitatively described the observed changes in cell volume and V_m_. Simulations offered complementary information that explain how the opening of K^+^ and Cl^−^ channels, as well as changes in their electrochemical gradients, account for the differences in the RVD responses observed in cells exposed to HYPO_NaCl_ versus HYPO_Mannitol_.

This observed participation of K^+^ and Cl^−^ channels in the RVD response of MIO-M1 cells is in line with previous reports in glial cells [4], [15], [32], [33]. It is interesting to note that in simulations, that only considered passive K^+^ and Cl^−^ fluxes as RVD mechanism, RVD_10_ magnitude in HYPO_NaCl_ was 34%, a value comparable to the experimental fraction of RVD_10_ inhibited by the use of BaCl_2_ and NPPB (∼32% and ∼25%, respectively). Then, the differences between absolute values of experimental and simulated RVD_10_ (∼70%), can be explained, at least in part, by the existence of other RVD mechanisms which are neither being inhibited by these drugs nor simulated. Indeed, organic osmolyte release during cell volume regulation has been widely described in glial cells [15], [34]. Nevertheless, NPPB is also a well known blocker of this RVD mechanism and is likely affecting organic osmolyte release in our experiments. In fact, some previous evidence showed that in glial cells a residual fraction of organic osmolyte release was observed even after NPPB inhibition [35]. Moreover, in hyppocampal slices, where an exocytosis-mediated mechanism was proposed, a fraction of organic osmolyte release was not sensitive to NPPB [36]. Another possibility is that part of the RVD response could be associated to KCl efflux by KCC co-transporters although in other systems, RVD rates mediated by this transporter are lower than those measured in our study [15]. This, however, does not rule out their potential contribution to the RVD response in MIO-M1 cells. Finally, it cannot be discarded that the technique used in our study to assess RVD could be overestimating the real cell volume changes. Nevertheless, the high RVD rates observed in the MIO-M1 cells in our work are very close to previously reported data in glial cells using other techniques [4], [15]. Although future studies are necessary to completely unmask RVD in MIO-M1 cells, it is likely that a combination of all the above described mechanisms explains the differences between experimental and simulated data.

Our data also demonstrated that, regardless of the activation of K^+^ and Cl^−^ channels, their contribution to the RVD response depends on the equilibrium potentials of these ions relative to the resting membrane potential of the cell. In fact, we found that the nature of the RVD response is affected by the steady-state condition previous to the osmotic shock. Our experiments indicated that, at steady-state, Eq_K_ is more negative relative to V_m_ while, as previously shown in glial cells [37], [38], Eq_Cl_ is less negative than the V_m_. MIO-M1 cells exposed to an isoosmotic solution in which NaCl was partially replaced with mannitol (ISO_Mannitol_) had a slightly more negative V_m_ than those exposed to a normal NaCl. When these cells are challenged with a hypoosmotic solution that keeps the same ionic strength, the intracellular compartment is diluted, thus reducing the chemical gradient of Cl^−^ and K^+^, a fact that opposes KCl efflux, and thus RVD. On the other hand, when cells are exposed to HYPO_NaCl_, the intracellular compartment is also diluted, but since the external solution has a reduced Cl^−^ concentration, the driving force for KCl efflux decreases to a lesser extent as compared to the HYPO_Mannitol_ condition. Therefore, the magnitude of KCl efflux during cell volume regulation will depend not only of the activation of specific ion channels but also of the magnitude of the driving forces of these ions. This also explains why experiments with HYPO_Mannitol_, in which KCl efflux is diminished, the blockage of K^+^ and Cl^−^ channels does not affect RVD response.

In our experiments, regardless of the composition of the hypoosmotic solution, cell swelling causes V_m_ depolarization. This depolarization could be at least partially explained by the dilution of intracellular K^+^, thus shifting Eq_K_ and V_m_ to less negative values and/or to the activation of channels corresponding to permeant species that exhibits an Eq positive to V_m_. We suggest that in MIO-M1 cells the opening of Cl^−^ channels certainly contributes to cell depolarization during swelling, as previously described in other cells types [38], [39]. In fact, the blockage of Cl^−^ channels in the HYPO_NaCl_ condition resulted in a markedly reduced swelling-induced depolarization. Nevertheless, it cannot be discarded that other ions with Eq that are more positive than V_m_ could also account for this depolarization, as reported in others cell types [40], [41], [42].

Our results revealed that the gradual V_m_ repolarization that coincides with RVD can be explained by the dissipation of Cl^−^ and K^+^ electrochemical gradients, which lead the cells to a new stationary-state. Clearly, the magnitude of this repolarization is lower in HYPO_Mannitol_, a condition in which these gradients are reduced as compared to HYPO_NaCl_. Even more, the blockage of K^+^ and Cl^−^ channels only in those conditions in which electrochemical gradients are considerable, like in HYPO_NaCl_, significantly reduces RVD and V_m_ repolarization by preventing the dissipation of these gradients.

Altogether, experimental and theoretical observations allow us to propose that the efficiency of the RVD process in Müller glia depends not only on the activation of ion channels, but is also strongly modulated by concurrent changes in the membrane potential. Thus, the relationship between ion permeability changes and volume regulation is complex and increments in K^+^ and Cl^−^ ionic conductances do not necessarily induce osmolyte fluxes large enough to give rise to RVD.

A better understanding of the relationship between cell volume and V_m_ in the central nervous system is of great interest, since neural activity itself as well as certain pathological conditions induce large alterations in extracellular fluid composition −particularly in Na^+^, K^+^ and Cl^−^ concentrations− which are associated to changes in these two interdependent baseline parameters, important for cellular function.

## References

[1] Kofuji P, Newman EA (2004) Potassium buffering in the central nervous system. Neuroscience 129: 1045–1056. 15561419 10.1016/j.neuroscience.2004.06.008 PMC2322935

[2] Bringmann A, Pannicke T, Grosche J, Francke M, Wiedemann P, et al (2006) Muller cells in the healthy and diseased retina. Prog Retin Eye Res 25: 397–424. 16839797 10.1016/j.preteyeres.2006.05.003

[3] Dmitriev AV, Govardovskii VI, Schwahn HN, Steinberg RH (1999) Light-induced changes of extracellular ions and volume in the isolated chick retina-pigment epithelium preparation. Vis Neurosci 16: 1157–1167. 10614595 10.1017/s095252389916615x

[4] Pasantes-Morales H, Murray RA, Lilja L, Moran J (1994) Regulatory volume decrease in cultured astrocytes. I. Potassium- and chloride-activated permeability. Am J Physiol 266: C165–171. 8304413 10.1152/ajpcell.1994.266.1.C165

[5] Vitarella D, DiRisio DJ, Kimelberg HK, Aschner M (1994) Potassium and taurine release are highly correlated with regulatory volume decrease in neonatal primary rat astrocyte cultures. J Neurochem 63: 1143–1149. 8051556 10.1046/j.1471-4159.1994.63031143.x

[6] Lang F, Busch GL, Ritter M, Volkl H, Waldegger S, et al (1998) Functional significance of cell volume regulatory mechanisms. Physiol Rev 78: 247–306. 9457175 10.1152/physrev.1998.78.1.247

[7] Hirrlinger PG, Wurm A, Hirrlinger J, Bringmann A, Reichenbach A (2008) Osmotic swelling characteristics of glial cells in the murine hippocampus, cerebellum, and retina in situ. J Neurochem 105: 1405–1417. 18221375 10.1111/j.1471-4159.2008.05243.x

[8] Wurm A, Pannicke T, Iandiev I, Wiedemann P, Reichenbach A, et al (2006) The developmental expression of K+ channels in retinal glial cells is associated with a decrease of osmotic cell swelling. Glia 54: 411–423. 16886204 10.1002/glia.20391

[9] Pannicke T, Iandiev I, Uckermann O, Biedermann B, Kutzera F, et al (2004) A potassium channel-linked mechanism of glial cell swelling in the postischemic retina. Mol Cell Neurosci 26: 493–502. 15276152 10.1016/j.mcn.2004.04.005

[10] Pannicke T, Uckermann O, Iandiev I, Wiedemann P, Reichenbach A, et al (2005) Ocular inflammation alters swelling and membrane characteristics of rat Muller glial cells. J Neuroimmunol 161: 145–154. 15748953 10.1016/j.jneuroim.2005.01.003

[11] Pannicke T, Iandiev I, Wurm A, Uckermann O, vom Hagen F, et al (2006) Diabetes alters osmotic swelling characteristics and membrane conductance of glial cells in rat retina. Diabetes 55: 633–639. 16505225 10.2337/diabetes.55.03.06.db05-1349

[12] Kuhrt H, Wurm A, Karl A, Iandiev I, Wiedemann P, et al (2008) Muller cell gliosis in retinal organ culture mimics gliotic alterations after ischemia in vivo. Int J Dev Neurosci 26: 745–751. 18672046 10.1016/j.ijdevneu.2008.07.003

[13] Olson JE, Li GZ (1997) Increased potassium, chloride, and taurine conductances in astrocytes during hypoosmotic swelling. Glia 20: 254–261. 9215734 10.1002/(sici)1098-1136(199707)20:3<254::aid-glia9>3.0.co;2-7

[14] Kimelberg HK, Anderson E, Kettenmann H (1990) Swelling-induced changes in electrophysiological properties of cultured astrocytes and oligodendrocytes. II. Whole-cell currents. Brain Res 529: 262–268. 2282496 10.1016/0006-8993(90)90836-z

[15] Ernest NJ, Weaver AK, Van Duyn LB, Sontheimer HW (2005) Relative contribution of chloride channels and transporters to regulatory volume decrease in human glioma cells. Am J Physiol Cell Physiol 288: C1451–1460. 15659714 10.1152/ajpcell.00503.2004 PMC2548409

[16] Wang K, Wondergem R (1992) Mouse hepatocyte membrane potential and chloride activity during osmotic stress. Am J Physiol 263: G566–572. 1415716 10.1152/ajpgi.1992.263.4.G566

[17] Ballanyi K, Grafe P, Serve G, Schlue WR (1990) Electrophysiological measurements of volume changes in leech neuropile glial cells. Glia 3: 151–158. 2141591 10.1002/glia.440030302

[18] Lewis R, Asplin KE, Bruce G, Dart C, Mobasheri A, et al.. (2011) The role of the membrane potential in chondrocyte volume regulation. J Cell Physiol. 10.1002/jcp.22646 PMC3229839 21328349

[19] Limb GA, Salt TE, Munro PM, Moss SE, Khaw PT (2002) In vitro characterization of a spontaneously immortalized human Muller cell line (MIO-M1). Invest Ophthalmol Vis Sci 43: 864–869. 11867609

[20] Ford P, Rivarola V, Chara O, Blot-Chabaud M, Cluzeaud F, et al (2005) Volume regulation in cortical collecting duct cells: role of AQP2. Biol Cell 97: 687–697. 15859948 10.1042/BC20040116

[21] Hamann S, Kiilgaard JF, Litman T, Alvarez-Leefmans FJ, Winther BR, et al (2002) Measurement of Cell Volume Changes by Fluorescence Self-Quenching. Journal of Fluorescence 12: 139–145.

[22] Klapperstuck T, Glanz D, Klapperstuck M, Wohlrab J (2009) Methodological aspects of measuring absolute values of membrane potential in human cells by flow cytometry. Cytometry A 75: 593–608. 19504578 10.1002/cyto.a.20735

[23] Brauner T, Hulser DF, Strasser RJ (1984) Comparative measurements of membrane potentials with microelectrodes and voltage-sensitive dyes. Biochim Biophys Acta 771: 208–216. 6704395 10.1016/0005-2736(84)90535-2

[24] Epps DE, Wolfe ML, Groppi V (1994) Characterization of the steady-state and dynamic fluorescence properties of the potential-sensitive dye bis-(1,3-dibutylbarbituric acid)trimethine oxonol (Dibac4(3)) in model systems and cells. Chem Phys Lipids 69: 137–150. 8181103 10.1016/0009-3084(94)90035-3

[25] Hernandez JA, Cristina E (1998) Modeling cell volume regulation in nonexcitable cells: the roles of the Na+ pump and of cotransport systems. Am J Physiol 275: C1067–1080. 9755060 10.1152/ajpcell.1998.275.4.C1067

[26] Poignard C, Silve A, Campion F, Mir LM, Saut O, et al (2011) Ion fluxes, transmembrane potential, and osmotic stabilization: a new dynamic electrophysiological model for eukaryotic cells. Eur Biophys J 40: 235–246. 21079946 10.1007/s00249-010-0641-8

[27] Ilyaskin AV (2011) Study of the Reaction of Kidney Collecting Duct Principal Cells to Hypotonic Shock. Experiment and Mathematical Model. Biophysics 56: 516–524.

[28] Hodgkin AL, Katz B (1949) The effect of sodium ions on the electrical activity of giant axon of the squid. J Physiol 108: 37–77. 18128147 10.1113/jphysiol.1949.sp004310 PMC1392331

[29] Goldman DE (1943) Potential, Impedance, and Rectification in Membranes. J Gen Physiol 27: 37–60. 19873371 10.1085/jgp.27.1.37 PMC2142582

[30] Lang F (2007) Mechanisms and significance of cell volume regulation. J Am Coll Nutr 26: 613S–623S. 17921474 10.1080/07315724.2007.10719667

[31] Eberhardt W, Reichenbach A (1987) Spatial buffering of potassium by retinal Muller (glial) cells of various morphologies calculated by a model. Neuroscience 22: 687–696. 3670605 10.1016/0306-4522(87)90365-4

[32] Wurm A, Lipp S, Pannicke T, Linnertz R, Farber K, et al (2009) Involvement of A(1) adenosine receptors in osmotic volume regulation of retinal glial cells in mice. Mol Vis 15: 1858–1867. 19756184 PMC2743807

[33] Pannicke T, Wurm A, Iandiev I, Hollborn M, Linnertz R, et al (2010) Deletion of aquaporin-4 renders retinal glial cells more susceptible to osmotic stress. J Neurosci Res 88: 2877–2888. 20544823 10.1002/jnr.22437

[34] Pasantes-Morales H, Murray RA, Sanchez-Olea R, Moran J (1994) Regulatory volume decrease in cultured astrocytes. II. Permeability pathway to amino acids and polyols. Am J Physiol 266: C172–178. 8304414 10.1152/ajpcell.1994.266.1.C172

[35] Pasantes-Morales H, Franco R, Ochoa L, Ordaz B (2002) Osmosensitive release of neurotransmitter amino acids: relevance and mechanisms. Neurochem Res 27: 59–65. 11926277 10.1023/a:1014850505400

[36] Franco R, Torres-Marquez ME, Pasantes-Morales H (2001) Evidence for two mechanisms of amino acid osmolyte release from hippocampal slices. Pflugers Arch 442: 791–800. 11512036 10.1007/s004240100604

[37] Kimelberg HK (1981) Active accumulation and exchange transport of chloride in astroglial cells in culture. Biochim Biophys Acta 646: 179–184. 6268162 10.1016/0005-2736(81)90285-6

[38] Kimelberg HK, O’Connor E (1988) Swelling of astrocytes causes membrane potential depolarization. Glia 1: 219–224. 2976041 10.1002/glia.440010307

[39] Parkerson KA, Sontheimer H (2003) Contribution of chloride channels to volume regulation of cortical astrocytes. Am J Physiol Cell Physiol 284: C1460–1467. 12606317 10.1152/ajpcell.00603.2002

[40] Galizia L, Flamenco MP, Rivarola V, Capurro C, Ford P (2008) Role of AQP2 in activation of calcium entry by hypotonicity: implications in cell volume regulation. Am J Physiol Renal Physiol 294: F582–590. 18094031 10.1152/ajprenal.00427.2007

[41] Welsh DG, Nelson MT, Eckman DM, Brayden JE (2000) Swelling-activated cation channels mediate depolarization of rat cerebrovascular smooth muscle by hyposmolarity and intravascular pressure. J Physiol 527 Pt 1: 139–148. 10.1111/j.1469-7793.2000.t01-1-00139.x PMC2270055 10944177

[42] Christensen O, Hoffmann EK (1992) Cell swelling activates K+ and Cl- channels as well as nonselective, stretch-activated cation channels in Ehrlich ascites tumor cells. J Membr Biol 129: 13–36. 1383549 10.1007/BF00232052

[43] Di Giusto G, Flamenco P, Rivarola V, Fernandez J, Melamud L, et al (2012) Aquaporin 2-increased renal cell proliferation is associated with cell volume regulation. J Cell Biochem 113: 3721–3729. 22786728 10.1002/jcb.24246

